# Host Cell Death and Modulation of Immune Response against *Mycobacterium tuberculosis* Infection

**DOI:** 10.3390/ijms25116255

**Published:** 2024-06-06

**Authors:** Annie Vu, Ira Glassman, Giliene Campbell, Stephanie Yeganyan, Jessica Nguyen, Andrew Shin, Vishwanath Venketaraman

**Affiliations:** College of Osteopathic Medicine of the Pacific, Western University of Health Sciences, Pomona, CA 91766, USAgilienegabrielle.cam@westernu.edu (G.C.); andrew.shin@westernu.edu (A.S.)

**Keywords:** *Mycobacterium tuberculosis*, apoptosis, necrosis, autophagy, cell death

## Abstract

*Mycobacterium tuberculosis* (*Mtb*) is the causative agent of tuberculosis (TB), a prevalent infectious disease affecting populations worldwide. A classic trait of TB pathology is the formation of granulomas, which wall off the pathogen, via the innate and adaptive immune systems. Some key players involved include tumor necrosis factor-alpha (TNF-α), foamy macrophages, type I interferons (IFNs), and reactive oxygen species, which may also show overlap with cell death pathways. Additionally, host cell death is a primary method for combating and controlling *Mtb* within the body, a process which is influenced by both host and bacterial factors. These cell death modalities have distinct molecular mechanisms and pathways. Programmed cell death (PCD), encompassing apoptosis and autophagy, typically confers a protective response against *Mtb* by containing the bacteria within dead macrophages, facilitating their phagocytosis by uninfected or neighboring cells, whereas necrotic cell death benefits the pathogen, leading to the release of bacteria extracellularly. Apoptosis is triggered via intrinsic and extrinsic caspase-dependent pathways as well as caspase-independent pathways. Necrosis is induced via various pathways, including necroptosis, pyroptosis, and ferroptosis. Given the pivotal role of host cell death pathways in host defense against *Mtb*, therapeutic agents targeting cell death signaling have been investigated for TB treatment. This review provides an overview of the diverse mechanisms underlying *Mtb*-induced host cell death, examining their implications for host immunity. Furthermore, it discusses the potential of targeting host cell death pathways as therapeutic and preventive strategies against *Mtb* infection.

## 1. Introduction

Tuberculosis (TB), caused by the bacillus *Mycobacterium tuberculosis* (*Mtb*), is a major public health concern today despite the prevalence of screening methods, vaccines, and the use of antibiotics. Although researchers have found that the number of deaths and those infected has continued to decrease over the past few decades, the immunocompromised and those in low-income countries continue to be the most at risk [1].

In 2022, per the latest data from the World Health Organization’s 2023 global tuberculosis report, approximately 10.6 million people fell ill with tuberculosis globally, which had increased from 10.3 million in 2021 and 10.0 million in 2020 [2]. TB caused around 1.6 million deaths in 2022, making it one of the top infectious disease killers worldwide [3]. The highest TB burden is in South East Asia, Africa, and the Western Pacific regions [2].

Acute TB typically presents slowly and nonspecifically with a persistent cough, chest pain, hemoptysis (coughing up blood), weight loss, fever, night sweats, and fatigue [4]. In immunocompromised individuals, such as those with HIV, acute TB can develop rapidly after initial infection [5]. Mtb is highly contagious in the acute phase, especially when symptoms are severe [6].

In chronic TB, also known as latent TB infection, individuals are often asymptomatic and are not contagious [7]. Mtb remains dormant for years or a lifetime without causing disease. Approximately 5–10% of latent infections progress to active TB disease, especially when the immune system is weakened. The reactivation of TB results in symptoms similar to those in acute TB, though they develop more gradually [8].

In terms of clinical disease burden, the complications of TB other than pulmonary symptoms include extrapulmonary dissemination and drug resistance. Pulmonary complications include cavitation, fibrosis, bronchiectasis, and lung impairment secondary to remodeling [9]. Extrapulmonary complications include miliary TB, TB meningitis, and TB pericarditis, which occur via the hematogenous spread of disseminated TB to multiple organs [10,11,12]. Additionally, multidrug-resistant TB (MDR-TB) can occur, where there is resistance to at least isoniazid and rifampin, the two most potent TB drugs [13].

*Mtb* infects alveolar macrophages and epithelial cells, which line the surface of the lungs and are transmitted by aerosol droplets when an infected individual coughs, sneezes, or talks [14]. *Mtb*’s entry into macrophages is accomplished through a lipid known as phthiocerol dimycocerosate (DIM), a major virulence factor [15]. After infection, *Mtb* is characteristically known to be “walled off” via the formation of granulomas [16,17]. However, this is not always successful in controlling infection, and *Mtb* has evolved multiple mechanisms to evade host immune responses [18].

Upon Mtb infection, the initial immune response involves important cytokines, such as tumor necrosis factor-alpha (TNF-α) and interferons (IFNs), specifically type II, which consists of a single member, IFN-γ. TNF is critical for activating macrophages, the primary cells involved in engulfing and attempting to kill M. tuberculosis [19]. Activated macrophages enhance their antimicrobial activity through the production of reactive nitrogen and oxygen intermediates, which can kill or inhibit the growth of the bacteria. TNF is essential for the formation and maintenance of granulomas, which are organized structures of immune cells that form around the bacteria to contain the infection, though they may also provide a niche where the bacteria can persist in a dormant state [19]. TNF promotes apoptosis and is a potent proinflammatory cytokine that recruits various immune cells, such as neutrophils and T cells, to the site of infection. While essential for containing the infection, excessive TNF production can lead to tissue damage and pathology associated with TB [20]. Furthermore, type II interferon IFN-γ, produced primarily by T cells and natural killer (NK) cells, is also crucial for the activation of macrophages, induction of antigen presentation by increasing the expression of major histocompatibility complex (MHC) molecules on macrophages, promoting the Th1 (T-helper 1) immune response, and promoting autophagy and enhancing the maturation of phagosomes, the vesicles in which macrophages contain ingested bacteria, leading to more effective fusion with lysosomes and destruction of the bacteria [21,22,23]. The coordinated action of TNF and IFN-γ is crucial for controlling TB infection; an insufficient response can lead to uncontrolled infection, while an excessive response can cause immunopathology.

Host cell death, a part of the innate immune response to microbial infection, is a significant mechanism in preventing the progression of *Mtb* infection via host–pathogen interactions [24]. Classically, the three types of cell death include apoptosis, autophagy, and necrosis [25]. New research has found new modes and pathways of cell death in recent years with the development of guidelines for categorizing cell death from morphological, biochemical, and functional perspectives [26,27]. Relevant non-apoptotic types of regulated cell death in *Mtb* infection include necroptosis, pyroptosis, and ferroptosis [27]. However, bacterial pathogens, including *Mtb*, have developed diverse strategies to manipulate host cell death and survival pathways, thereby enhancing their own replication and persistence [24]. Central to these strategies is the modulation of critical host cell pathways, including those governing mitochondrial pro-death signaling, nuclear factor-kappa B (NF-κB)-dependent pro-survival mechanisms, and inflammasome-dependent pathways [24].

Cell death of the *Mtb*-infected phagocyte most commonly occurs via apoptotic and non-apoptotic mechanisms. Apoptosis maintains an intact plasma membrane, whereas necrosis results in cell lysis, allowing *Mtb* to exit the phagocyte. As such, *Mtb* survival involves evading apoptosis and inducing necrosis [28]. Additionally, *Mtb* may take advantage of other innate and adaptive immune responses, such as autophagy and reactive oxygen species (ROS), to increase its virulence [29].

These findings are significant because they help to identify the factors correlated with the modulation of immune response by *Mtb* and also serve to identify targets for potential therapies. Although researchers have discovered specific factors contributing to *Mtb* virulence, confirming the mechanisms in which the human body responds to *Mtb* infections and *Mtb*’s evasion of the immune system will allow researchers to target specific organs and aspects of the immune system, leading to future treatments and potential vaccines. This article offers a general review on the modulation of immune responses and host cell death pathways involved in *Mtb* infections, as well as the ways in which *Mtb* subverts and evades these mechanisms, and discusses current therapies and future suggestions for host-directed therapies.

## 2. Immune System Modulation

Immune modulation refers to the dynamic process by which the host immune system regulates and adjusts its responses to pathogens. During *Mtb* infection, immune modulation involves the activation and regulation of various immune cells and signaling pathways to mount an effective defense against the pathogen while minimizing immunopathology. This includes the activation of macrophages, dendritic cells, T cells, and other immune cells, as well as the production of cytokines and chemokines that coordinate the immune response. Immune modulation aims to balance the need for pathogen clearance with the avoidance of excessive inflammation and tissue damage, thereby promoting host survival [30].

### 2.1. Innate and Adaptive Immune System

Host immune systems have a variety of responses to fight *Mtb* infections. To begin, the innate immune system has proven to be foundational against *Mtb*. In studies where the innate immune system has been knocked out, *Mtb* has proven to be fatal even with a significant adaptive immune response [31]. Thus, the innate immune system is key to understanding the pathogenesis of *Mtb*. The first defense against *Mtb* is alveolar macrophages. *Mtb* in its early stages is found primarily within alveolar macrophages and then is relocalized to the lung interstitium by IL-1 [32]. These alveolar macrophages phagocytose and degrade *Mtb*, containing it to prevent dissemination. However, *Mtb* has developed several protective measures to not only avoid degradation but proliferate within alveolar macrophages. Upon phagocytosis, *Mtb* inhibits the maturation of phagosome and inhibits phagolysosome formation [33]. Furthermore, if phagolysosome formation persists, *Mtb* is not degraded. The cellular endosome is unable to fuse with the phagolysosome, and this inhibits the acidification of *Mtb* [34]. In the phagolysosome, *Mtb* not only persists, but after several days, it is able to proliferate within the phagolysosome. One study by Houben et al. found that pathogenic *Mtb* can translocate to the cytosol to secrete 6 kDa early secretory antigenic target (ESAT-6) for cytosolic translocation via the ESAT-6 secretion system (ESX-1) [35].

Thymus-derived lymphocytes (T cells) bearing clusters of differentiation 4 glycoprotein (CD4+) are key to launching an effective adaptive immune response. Both alveolar macrophages and dendritic cells are antigen-presenting cells to activate CD4+ T cells after reaching the pulmonary lymph nodes [36]. In the same study, earlier dissemination of *Mtb* to the lymph nodes generated a faster T cell response. Furthermore, the faster the activation of CD4+ T cells, the greater the inhibition of *Mtb* [37]. However, *Mtb* has developed several mechanisms to inhibit T cell activation. During *Mtb* infection, the main antigen-presenting cells (alveolar macrophages and dendritic cells), once infected, have impaired recruitment to the lungs and migration to lymph nodes [38]. Furthermore, infected dendritic cells have been shown to have impaired CD4 T cell activation [39]. Infected macrophages similarly have decreased capacity for CD4+ T cell activation through decreased MHC II expression [40].

The importance of CD4+ T cells against *Mtb* infection is shown through *Mtb* and HIV coinfection. The depletion of appropriate CD4+ T cell responses in these studies led to an increased risk for active TB infection [41]. The direct activation of CD4 T cells rather than chemokine production leads to the control of intracellular *Mtb* [42]. Once activated, Th1 T cells produce interferon (IFN) gamma, which is key for resistance to *Mtb* infection [43]. CD4+ T cells have been shown to stimulate CD8 T cell IFN gamma production and cytotoxicity as well as the cytotoxicity of natural killer (NK) cells against *Mtb* [44].

There are various cytokines that are key to both the innate and adaptive immune response to *Mtb*. Granulocyte macrophage colony-stimulating factor (GM-CSF) controls macrophage survival and differentiation [45]. IL-12p40 is key for the migration of dendritic cells to the lymph to prime CD4 T cells [46]. CD4 T cells were found to produce multiple proinflammatory cytokines such as IFN gamma, TNF-α, and IL-2 that provide protective effects but also induce many of the damaging effects of *Mtb* during active infection [47].

### 2.2. TNF-α and Granuloma Formation

Of note, TNF-α is essential for the induction of programmed cell death (apoptosis) in infected cells, a process crucial for limiting the intracellular replication of *Mtb* and facilitating its clearance by the immune system.

After persistent *Mtb* infection, the host moves to limit the infection through the formation of granulomas. To wall off the infection, monocytes and macrophages develop into epithelial macrophages that intertwine [48]. The development and maintenance of the granuloma are highly dependent on TNF-α for the recruitment of macrophages [49]. CD4 T cells were found to be key to completely closing off lesions and inhibiting *Mtb* dissemination from macrophages [50]. The granuloma serves to wall off infection but also allows for the survival of *Mtb* through the expression of various metabolic genes [51]. However, *Mtb* is able to persist in the granuloma with the help of foamy macrophages to provide nutrients and ultimately spreads through the cavitation of these foamy macrophages [52]. Cavitation is an unsuccessful containment of *Mtb* that leads to the spread of *Mtb*. Successful granuloma formation or unsuccessful cavitation is dependent on the structural organization of the granuloma [53]. In the same study, vascularization and the continuous proliferation of the immune response were key to containing *Mtb*. Another study arrived at the same conclusion, wherein cavitation was preceded by a depletion of CD4 and clusters of differentiation 8 glycoprotein (CD8) T cells [54]. Therefore, the successful inhibition of *Mtb* spread and inhibition is dependent on both successful innate and adaptive immune responses.

Excessive or dysregulated production of TNF-α can also contribute to immunopathology in tuberculosis. High levels of TNF-α can lead to tissue damage and inflammation, contributing to the pathology associated with tuberculosis, including the formation of caseous necrosis and lung damage. Moreover, TNF-α blockade or deficiency has been linked with the reactivation of latent tuberculosis infection and increased susceptibility to disseminated disease [49].

### 2.3. Role of Autophagy in Mtb Infection

Autophagy is a conserved cellular process crucial for maintaining cellular homeostasis by eliminating damaged organelles, misfolded proteins, and intracellular pathogens, which are engulfed by lysosomes for degradation [55]. Lysosomes break down these substrates and either recycle or create new sources of energy with the basic components [55]. Autophagy is triggered under stress conditions, such as caloric restriction, and as a defense against intracellular pathogens [56,57].

In the context of *Mtb* infection, autophagy plays a multifaceted role in innate and adaptive immune responses against the pathogen, including modulating proinflammatory cytokine production [58]. Host cells have pattern recognition receptors (PRRs) such as Toll-like receptors (TLRs) and nucleotide-binding oligomerization domain (NOD)-like receptors (NLRs) that recognize *Mtb*-associated molecular patterns (MAMPs) and trigger autophagy activation [59] as part of the innate immune response to control bacterial replication and promote antigen presentation [60]. Various signaling pathways, including the mammalian target of rapamycin (mTOR) and AMP-activated protein kinase (AMPK) pathways, are involved in the regulation of autophagy induction during *Mtb* infection [61].

Autophagy targets intracellular *Mtb* for degradation within autophagosomes, leading to bacterial killing and antigen presentation to activate adaptive immune responses [62]. Autophagic machinery components, such as autophagy-related proteins (ATGs) and LC3 (microtubule-associated protein 1A/1B-light chain 3), are recruited to *Mtb*-containing phagosomes to initiate autophagosome formation and engulf the bacteria [63,64]. Subsequently, autophagosomes fuse with lysosomes to form autolysosomes, where *Mtb* is degraded by lysosomal hydrolases [65].

**Figure 1:**
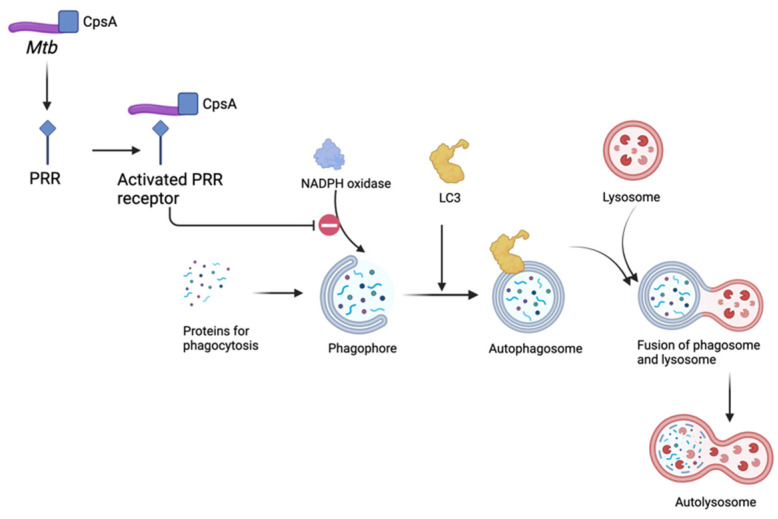
CpsA protein in LC3-associated phagocytosis. In LC3-associated phagocytosis, NADPH oxidase is recruited to the phagosome. There is a subsequent recruitment of LC3 and maturation of the phagophore, followed by a fusion of the subsequent autophagosome with a lysosome containing degradative enzymes, resulting in an autolysosome. The CpsA protein of *Mtb* may activate PRR but will inhibit the recruitment of NADPH oxidase.

Despite being targeted for autophagic clearance, *Mtb* has evolved various strategies to subvert or evade host autophagy mechanisms [29]. Virulent strains of *Mtb* can inhibit autophagy by interfering with signaling pathways or blocking autophagosome–lysosome fusion, allowing the bacteria to survive and replicate within host cells [66]. For example, LC3-associated phagocytosis (LAP) is a macrophage pathway which eliminates microbes by the recruitment of nicotinamide adenine dinucleotide phosphate (NADPH) oxidase to phagosomes, followed by phagosome association with LC3 and delivery of the bacteria to a degradative lysosome [67]. Koster et al. discovered that *Mtb* contains possible conserved protein A (CpsA), a member of the lytic repressor (LytR)-CpsA-PBP 5 synthesis repressor (Psr) (LCP) family involved in cell wall metabolism, which offers protection from LAP and ultimately prevents the successful clearance of *Mtb*, as seen in Figure 1 [68]. Furthermore, *Mtb* can actively inhibit autophagy by secreting virulence factors such as ESAT-6 (early secreted antigenic target 6) and CFP-10 (culture filtrate protein 10), which disrupt autophagic flux and impair bacterial clearance [69].

In addition to its direct antimicrobial effects, autophagy modulates host immune responses to *Mtb* infection by regulating inflammation, antigen presentation, and cytokine production. Autophagy-mediated degradation of *Mtb*-derived antigens enhances major histocompatibility complex (MHC) class II presentation to CD4+ T cells, promoting adaptive immunity against the pathogen [70]. Moreover, autophagy suppresses excessive inflammation by selectively targeting inflammasome components for degradation, thereby dampening cytokine release and mitigating immunopathology [71].

### 2.4. Foam Cell Macrophages and Mtb-Mediated Modulation

Macrophage-derived foam cells are filled with lipid droplets from the increased uptake of extracellular lipids from dead cells during *Mtb* infection [72,73]. These foam cells are observed in *Mtb* granulomas [72,73]. Histologically, *Mtb* foam cells are similar to those in atherosclerosis, though they distinctly have increased triglycerides relative to cholesterol [73]. Foam cells also produce eicosanoids, a lipid-based signaling molecule including prostaglandins, leukotrienes, and thromboxane [74,75]. An in vivo study in mice by Sorgi et al. further investigated the role of eicosanoids, in particular leukotriene and prostaglandins. While there were increased amounts of prostaglandin E2 (PGE2) detected during *Mtb* infection, the pharmacological inhibition of COX-2 induced a significant reduction in bacterial load via increased NO release and IFN-γ production and increased macrophage phagocytosis of *Mtb*. Additionally, a lack of the 5-lipoxygenase (5-LO) pathway necessary for producing leukotrienes was associated with *Mtb* resistance, and leukotriene B4 (LTB4) inoculation in mice without 5-LO increased susceptibility to *Mtb* infection [76]. This study implied that while PGE2 and LTB4 have proinflammatory effects necessary to combat *Mtb*, a fine balance is important to prevent severe inflammation that may shift the cell toward necrosis and ultimately *Mtb* dissemination [76]. Nore et al. further confirmed this with a study that showed the plasma levels of eicosanoids appearing to fluctuate across different clinical states of *Mtb* infection [77].

### 2.5. Role of Type I Interferons

Interferons (IFNs) are a group of signaling proteins that play a critical role in the immune response to infections, though not all types of interferons are equally involved in the response to Mtb infection due to their distinct mechanisms of action and the unique ways they modulate the immune system [22,78]. It is well known that IFN-γ, a type II interferon, is crucial for activating macrophages and is ultimately a protective function against *Mtb* [79]. In contrast, type I interferons (IFNs), including IFN-α and IFN-β, are pleiotropic cytokines that play a crucial role in the host response to viral infections [80,81]. However, emerging evidence suggests that type I IFNs may impair the immune response to *Mtb* infection and promote *Mtb* infection, albeit with complex and context-dependent effects [82,83].

A transcriptomic study in 2010 conducted among patients with active *Mtb* disease and individuals either latently infected or healthy in the UK and South Africa revealed type I IFN-inducible genes [84]. Specifically, there is an upregulation of IFN response genes, including STAT1 [82], IFITs [85], GBPs [86], MX1 [87], OAS1 [88], IRF1 [89], and others, in individuals progressing from tuberculosis exposure to active disease [82,90,91,92].

Sustained or dysregulated production of type I IFNs during chronic *Mtb* infection can have detrimental effects on the host immune response [93]. Excessive type I IFN signaling has been linked with immunopathology, including impaired macrophage function, dysregulated cytokine production, and altered T cell responses [93]. Specifically, the heightened production of type I IFN has been linked to the virulence of *Mtb* strains and heightened host vulnerability. Investigations into infections involving hypervirulent clinical isolates of *Mtb*, such as HN878 and BTB 02-171, in comparison to the less virulent laboratory strain (H37Rv), have demonstrated a direct association between elevated levels of type I IFN and heightened mycobacterial virulence [94,95,96]. This dysregulated immune response can contribute to the persistence of *Mtb*, exacerbating disease severity and promoting tissue damage [82].

**Figure 2:**
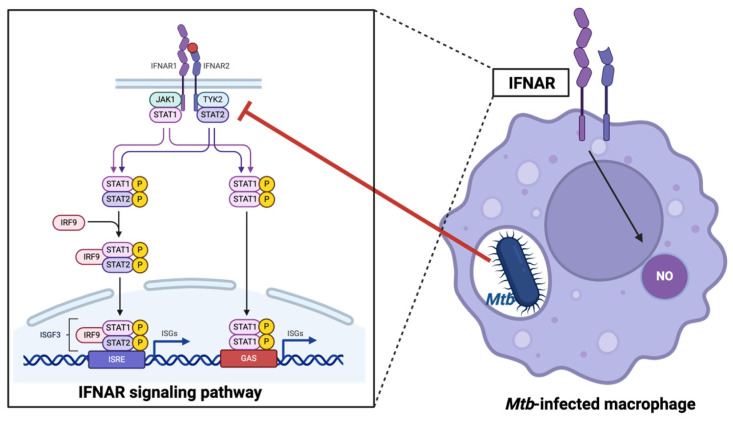
*Mtb* inhibition of IFN-associated receptor (IFNAR) signaling pathway, preventing nitric oxide production.

A recent study showed that IFN-β signaling in mouse macrophages has a protective effect against *Mtb* by increasing the production of nitric oxide, which can inhibit bacterial growth. However, *Mtb* has evolved mechanisms to inhibit type I IFN signaling in infected cells, likely as a strategy to evade host defense mechanisms [97], highlighting the intricate interplay between the host immune response and *Mtb* evasion strategies during infection. This protection is mediated by direct signaling through the IFN-associated receptor (IFNAR), as blocking IFNAR1 prevents nitric oxide production [97]. *Mtb* inhibits IFN-α/β receptor-mediated signaling and the transcription of 309 IFN-β stimulated genes in a dose-dependent manner. This inhibition involves reduced phosphorylation of IFNAR-associated kinases JAK1 and TYK2, leading to decreased phosphorylation of downstream targets STAT1 and STAT2 [97], as seen in Figure 2. *Mtb*-mediated inhibition of type I IFN signaling occurs only in infected cells, suggesting an evasion of host defense mechanisms.

## 3. Host Cell Death Pathways in *Mtb* Infection

Once the bacteria have been phagocytosed and are able to survive the host immune response, a cascade of reactions can occur, leading to various outcomes. The process of phagocytosis is mediated by receptors on host macrophages’ surfaces, such as macrophage mannose receptors and complement receptors [98]. These receptors have been shown to be requirements necessary for the beginning of the intracellular pathway of *Mtb* [98].

### 3.1. Apoptosis: In Favor of the Host

Apoptosis, or programmed cell death, is a highly regulated process essential for maintaining tissue homeostasis and eliminating infected or damaged cells. Researchers have identified host macrophage apoptosis as a key protective factor in innate immune response, restricting the growth of *Mtb* through deprivation of the bacteria’s niche cell, eliminating an environment conducive for bacterial growth and replication, and facilitating the clearance of infected cells [99]. The apoptosis of infected macrophages is commonly considered to be most likely beneficial to the host cells and can reduce the viability of *Mtb* [100], though another hypothesis proposes that it is most beneficial in the early stages of *Mtb* infection [101]. In an in vitro study investigating Fas-mediated apoptosis, it was observed that the controlled apoptotic event of macrophages led to a significant reduction in bacterial viability [100]. Apoptotic cells are rapidly engulfed and cleared by phagocytes via a process called efferocytosis, preventing the release of intracellular *Mtb* and minimizing inflammation and tissue damage [102,103]. In particular, *Mtb* is only eradicated following efferocytosis, suggesting that apoptosis alone does not inherently eliminate the bacterium but necessitates subsequent phagocytic ingestion and lysosomal fusion of the apoptotic body containing the bacterium [103].

### 3.2. Caspase-Dependent Pathways

**Figure 3:**
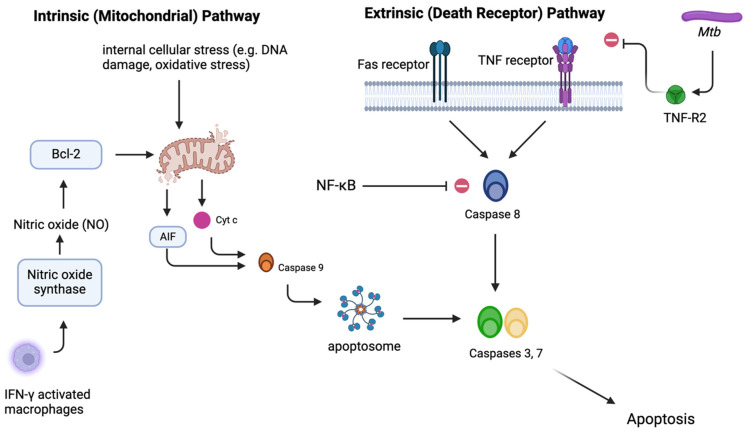
Caspase-dependent apoptosis may occur via the intrinsic or extrinsic pathway. The intrinsic pathway is triggered by internal cellular stress or by proapoptotic Bcl-2 proteins, which cause the release of factors such as cytochrome c and apoptosis-inducing factor (AIF) from the mitochondrial intermembrane space. Such factors activate caspase 9, which activates the apoptosome, which further activates caspase 3 or 7. Of note, nitric oxide (NO) is found to upregulate Bcl-2 proteins. IFN-γ-activated macrophages further upregulate nitric oxide synthase, thereby increasing NO and thus proapoptotic Bcl-2 proteins. In contrast, the extrinsic pathway is activated via death receptors, such as Fas or TNF receptor. This activates caspase 8, which further activates caspase 3 or 7. Of note, *Mtb* may secrete TNF-R2, which can inhibit the TNF receptor.

*Mtb*-induced apoptosis can be triggered through various signaling pathways, including intrinsic and extrinsic pathways, both of which converge at the activation of caspases, ultimately resulting in cell death via apoptosis [104]. The intrinsic pathway, also known as the mitochondrial pathway of apoptosis, is initiated by internal cellular stress signals such as DNA damage, oxidative stress, or growth factor withdrawal. Consequent cytotoxic stimuli and proapoptotic signal-transducing molecules, such as those of the Bcl family, induce permeability in the outer mitochondrial membrane [105,106]. Upon disruption of the outer mitochondrial membrane, various proteins found in the mitochondrial intermembrane space are released into the cytoplasm, most notably cytochrome c [107], triggering the formation of the apoptosome, a protein complex that activates caspase enzymes [108]. Another notable protein released is apoptosis-inducing factor (AIF), which provokes the release of caspase 9 [109]. This activation cascade ultimately leads to the cleavage of cellular components, DNA fragmentation, and cell death, as seen in Figure 3.

Snyder et al. showed that nitric oxide (NO) also activates the intrinsic pathway of apoptosis, specifically by activating Bcl-2 proteins BAX and BAK and releasing cytochrome c from the mitochondria [110,111]. Additionally, Herbst et al. showed that IFN-γ-activated macrophages upregulated the expression of nitric oxide synthase (NOS2) and exposes bacteria to nitric oxide (NO) via the caspase 3 and 7 pathways, allowing IFN-γ-induced apoptosis, as seen in Figure 3 [112].

In contrast, the extrinsic pathway, also known as the death receptor pathway of apoptosis, is initiated by external signals binding to death receptors on the cell surface, such as Fas ligand (FasL), tumor necrosis factor alpha (TNF-α), tumor necrosis factor-related apoptosis-inducing ligand RI (TRAIL-RI), and TRAIL-RII [100,104,113]. Binding of the ligand to the death receptor activates initiator caspase 8, which can directly cleave downstream effector caspases such as caspase 3 and, ultimately, cellular substrates and apoptosis, as seen in Figure 3 [114].

A study by Kelly et al. showed evidence of bystander macrophage apoptosis contributing to the innate immune response against *Mtb,* wherein infected macrophages induce the apoptosis of uninfected macrophages via a cell-to-cell contact-dependent mechanism [115]. There was no evidence of typical intrinsic or extrinsic pathway involvement of TNF- a, Fas, tumor necrosis factor-related apoptosis-inducing ligand, transforming growth factor β, Toll-like receptor 2, or MyD88 in contact-mediated bystander apoptosis [115]. Following cell death, antigen-presenting cells release extracellular vesicles that carry mycobacterial antigens to uninfected bystander cells to activate CD8 T cells that can recognize and lyse infected cells [116].

There are various studies showing that *Mtb* may both induce and inhibit apoptosis, which may be influenced by the viability of bacteria and host cells as well as the timing of infection [18]. *Mtb*’s ability to modulate apoptosis was seen in an in vitro study that showed different virulent strains inducing different levels of apoptosis. Specifically, virulent forms of *Mtb* modulated and reduced the occurrence of apoptosis, whereas more apoptosis occurred in avirulent or attenuated strains [117]. *Mtb* can secrete several proteins that inhibit apoptosis [18].

For example, *Mtb* may evade apoptosis by the release of TNF-R2 via an IL-10-dependent mechanism, resulting in the inactivation of TNF-α, as seen in Figure 3 [118,119,120]. TNF-α, a proinflammatory cytokine, has been identified as a factor that is associated with attenuated *Mtb* strains and apoptosis [118]. One piece of research showed that mice treated with a monoclonal antibody against TNF-α and those deficient in the TNF-α receptor developed lethal *Mtb* infections due to cell necrosis in comparison to controls [121]. Additionally, *Mtb* induces IL-10 production in macrophages, which further downregulates TNF-a production [119,120]. IL-10 is an anti-inflammatory cytokine associated with reduced CD4+ T cell response in *Mtb* infection [122,123]. IL-10 has also been found to decrease cytokine response in lymph nodes and lung granulomas, as well as reducing collagenization and fibrosis, affecting the structure of granulomas and demonstrating IL-10’s significant role in the modulation of the immune response and *Mtb*’s evasion of the adaptive immune system [124]. Furthermore, research shows that antigen-specific CD4+ T cells are activated once *Mtb* is in the mediastinal lymph nodes, despite the fact that there are a much larger number of bacteria in the lungs. These results allowed researchers to conclude that the delay in the activation of CD4+ T cells occurs due to a delay in the transport of *Mtb* from the lungs to the lymph nodes [125].

*Mtb* can also evade apoptosis by taking advantage of antiapoptotic mechanisms in the intrinsic apoptosis pathway [126,127,128]. Members of the B cell lymphoma 2 (BCL-2) family regulate apoptosis via the inhibition and induction of cell death [128,129]. They directly bind and regulate mitochondrial outer membrane permeability, leading to the release of proteins from the intermembrane space necessary for initiating the caspase cascade and ultimately apoptosis [130]. NF-κB has antiapoptotic activity and its activation inhibits death receptor-activated caspases [131]. As such, a study by Dhiman et al. found that the *Mtb* activation of NF-κB led to the upregulation of the BCL-2 antiapoptotic member bfl-1A1 [126]. Another study by Sly et al. found that virulent strains of *Mtb* upregulated and induced Mcl-1 expression [127,132].

Conversely, *Mtb* may facilitate host cell apoptosis via surface proteins or secreted proteins. For example, *Mtb* Rv3261 protein inhibits *Mtb* growth in macrophages and induces ROS production, which activates the caspase 3/9-dependent pathway and leads to macrophage apoptosis [133]. An in silico analysis study conducted by Medha et al. showed that *Mtb* PE6 (Rv0335c) had similar C-terminal and N-terminal sequences to Bcl2 proteins [134]. In vitro experiments further confirmed that Rv0335c’s molecular similarity to host Bcl2 proteins likely allows for host mitochondrial perturbations and apoptosis. A proposed hypothesis is that apoptosis during late-stage infection during granuloma formation might be a pathogenic process that increases *Mtb*’s persistence and future dissemination [101].

### 3.3. Caspase-Independent Pathways

Apoptosis during *Mtb* infection can occur through various caspase-independent pathways, including ROS induction, mitochondrial dysfunction, and endoplasmic reticulum (ER) stress [135].

ROS play a complex role in *Mtb* infection, acting as both a host defense mechanism and a target for bacterial evasion strategies [136]. ROS-induced oxidative stress can activate antimicrobial defense pathways within host cells, leading to the induction of autophagy and programmed cell death (apoptosis) to eliminate intracellular *Mtb* [137]. Furthermore, upon encountering *Mtb*, immune cells such as macrophages and neutrophils undergo a respiratory burst, resulting in the rapid production of ROS [138]. ROS exert direct antimicrobial effects by damaging essential macromolecules such as lipids, proteins, and DNA within *Mtb*. Furthermore, ROS can disrupt *Mtb*’s redox balance, impairing its metabolic activity and promoting bacterial killing [139].

**Figure 4:**
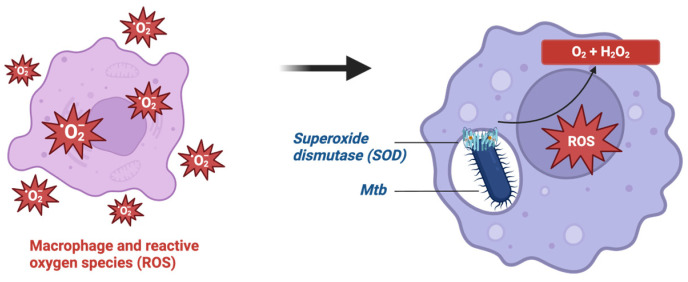
Antioxidant defense in *Mtb* via superoxide dismutase.

Despite the bactericidal effects of ROS, *Mtb* has evolved various strategies to counteract oxidative stress and survive within the hostile intracellular environment. Firstly, *Mtb* possesses antioxidant defense mechanisms, including enzymes such as catalase-peroxidase (KatG) and superoxide dismutase (SOD) (Figure 4), which detoxify ROS and protect the bacterium from oxidative damage [140]. It has been reported that *Mtb* can evade host immunity by binding to triggering receptor expressed on myeloid cells (TREM2), a macrophage surface receptor that mediates anti-inflammatory immune signaling. This promotes the intracellular survival of *Mtb* by inducing type I IFN signaling and suppressing the production of ROS [45]. In another study, Afriyie et al. found that *Mtb* can inhibit ROS production and induce necrotic cell death by reducing the expression of focal adhesion kinase (FAK), which led to necrotic cell death and persistent lung damage [141]. The study used pharmacological interventions to inhibit FAK, which increased necrosis due to *Mtb*. In contrast, genetic overexpression of FAK prevented cell death during *Mtb* infection [141].

As previously mentioned above regarding the intrinsic pathway of apoptosis, mitochondrial damage releases apoptosis-inducing factor (AIF), which triggers chromatin condensation and DNA fragmentation [142]. Initial studies show the translocation of AIF from the mitochondria to the cytosol and ultimately the nucleus, where it facilitates chromatin condensation [135].

Stress on the endoplasmic reticulum (ER) can result in the accumulation and aggregation of unfolded proteins, which trigger apoptosis via the unfolded protein response [143,144,145]. Surprisingly, live *Mtb* H37Rv can induce an ER stress response, implying that the ER stress pathway also contributes to the persistence and pathogenesis of *Mtb* [146]. A recent study by Xu et al. discovered a novel *Mtb* protein CDP-diglyceride hydrolase (Cdh), which interacted with the endoplasmic reticulum and led to cell death [147].

## 4. Necrosis: In Favor of *Mtb*

Necrosis, in contrast to apoptosis, occurs due to cytotoxic signals that result in the swelling of cell organelles, plasma membrane rupture, and the eventual lysis of the cells, leading to the release of proteins that contribute to inflammasome activation and the secretion of proinflammatory cytokines [148]. Although traditionally considered a passive and unregulated form of cell death while apoptosis was considered the regulated form of cell death, emerging evidence suggests that necrosis can be induced by specific signaling pathways and molecular mechanisms, a process termed “regulated necrosis” or “necroptosis” [149].

During *Mtb* infection, necrosis can be induced through various pathways, including necroptosis, pyroptosis, and ferroptosis. Necroptosis is a form of regulated necrosis mediated by receptor-interacting protein kinases (RIPKs) and mixed-lineage kinase domain-like protein (MLKL), leading to plasma membrane rupture and the release of damage-associated molecular patterns (DAMPs) that exacerbate inflammation and tissue injury [149]. Pyroptosis, mediated by inflammasome activation and caspase 1 activation, leads to rapid cell lysis and the release of proinflammatory cytokines, promoting host defense but also contributing to immunopathology [150]. Ferroptosis, characterized by iron-dependent lipid peroxidation and oxidative stress, represents a novel form of regulated necrosis implicated in *Mtb* pathogenesis [151].

Virulent strains of *Mtb* interfere with the progression of apoptosis and instead facilitate necrosis to necrosis [152]. Investigation of the interaction between *Mtb* and macrophages finds that three distinct mechanisms contribute to macrophage necrosis. First, *Mtb* inhibits plasma membrane repair [153]. Second, virulent *Mtb* causes inner mitochondrial membrane damage [152]. Third, virulent *Mtb* inhibits the cross-linking of annexin-1, which impairs formation of the apoptotic envelope [28]. This proves to have significant outcomes as necrotic macrophages provide *Mtb* with an environment that is best suited for replication [154]. Ultimately, macrophage necrosis results in increased replication, dissemination, and virulence in *Mtb*.

## 5. Therapeutic Considerations Targeting Autophagy, Apoptosis, and Necrosis in *Mtb* Infection

Several pharmacologic agents have been identified for their ability to target autophagy to promote antimicrobial effects against *Mtb* infection. For example, the use of small molecules or compounds that activate autophagy pathways, such as rapamycin, an mTOR inhibitor, has been shown to induce autophagy and enhance the killing of *Mtb* in infected macrophages [155]. Metformin, an activator of AMP-activated protein kinase (AMPK) and regulator of the autophagy pathway, has been shown to induce autophagy, even in cases of *Mtb* drug resistance [61,156]. Transcription factors such as TFEB (transcription factor EB) regulate the expression of genes involved in autophagy and lysosomal biogenesis seen in *Mtb*. Other drugs investigated for their potential in anti-Tb therapy and inducing autophagy include histone deacetylase (HDAC) inhibitors [157], lysosomotropic agents [158], which accumulate in acidic compartments such as lysosomes and may enhance the autophagic clearance of intracellular *Mtb*, and the antiprotozoal drug nitazoxanide and its analogs, which activate autophagosome formation and mTORC1 inhibition [159]. Vitamin D has also demonstrated efficacy in reducing *Mtb* load through synthesis of the autophagic antimicrobial peptide LL-37, the active form of cathelecidin antimicrobial peptide (CAMP) [160].

Targeting apoptosis as a therapeutic strategy for *Mtb* infection is an active area of research, with several potential therapies under investigation. For example, compounds that can directly induce apoptosis in *Mtb*-infected cells may have therapeutic potential, such as BCL-2 homology domain 3 (BH3) mimetics, which mimic the action of proapoptotic proteins [161]. For *Mtb* strains which have evolved mechanisms to inhibit host cell apoptosis as a means of promoting their survival, targeting antiapoptotic pathways may enhance the clearance of *Mtb*-infected cells, as seen with cellular inhibitors of apoptosis proteins (cIAPs), which suppress apoptosis [162,163]. Host factors that regulate apoptosis can be targeted for therapeutic intervention. p53, a tumor suppressor protein that plays a role in apoptosis, has been implicated in the host response to *Mtb* infection. Lim et al. found that nutlin-3, a p53 activator, significantly suppressed the intracellular survival of *Mtb* in hosts, suggesting another potential therapeutic target for *Mtb* therapy [164]. Therapies aimed at modulating the host immune response to *Mtb* infection may indirectly influence apoptosis and promote bacterial clearance. For example, cytokine-based therapies or immune checkpoint inhibitors could enhance the ability of the immune system to eliminate *Mtb*-infected cells through apoptosis, though there have been several reported cases of severe or reactivated *Mtb* in cancer patients [165,166].

Targeting necrosis as a therapeutic strategy for *Mtb* infection is also undergoing exploration, although specific therapies directly targeting necrosis are limited. Since necrosis is often associated with excessive inflammation, anti-inflammatory agents may help mitigate necrosis-associated tissue damage and inflammation during *Mtb* infection. For example, corticosteroids or non-steroidal anti-inflammatory drugs (NSAIDs) may be used as adjunctive therapy to reduce inflammation and tissue damage caused by necrosis [167]. NSAIDs and corticosteroids, among other therapies modulating eicosanoids such as the 5-lipoxygenase inhibitor zileuton and amine tricyclic antidepressant desipramine, have also demonstrated an ability to reduce necroptosis by impeding the arachidonic acid pathway [168]. This pathway is utilized by virulent strains of *Mtb* through the production of lipoxin A_4_, which favors necrosis by inhibiting prostaglandin E_2_, an essential cellular membrane maintenance eicosanoid. The arachidonic acid pathway also leads to increased TNF, a necroptotic cytokine favoring high inflammation states such as *Mtb* infection, which can be targeted by phosphodiesterase (PDE) inhibitors [169], specifically the PDE4 inhibitor CC-11050, which has demonstrated an enhancement of isoniazid antimycobacterial activity in animal models [169].

While necrosis itself is not directly targeted by antimicrobial agents, the effective treatment of *Mtb* infection with antibiotics may help prevent or reduce necrosis by controlling bacterial replication and limiting tissue damage. First-line anti-tuberculosis drugs, such as isoniazid, rifampicin, and pyrazinamide, are essential components of standard TB treatment regimens and play a crucial role in controlling *Mtb* growth and spread [170]. Unfortunately, standard antibiotic therapy involves expensive long-term treatment and side effects and has led to the development of multi-drug-resistant TB [171]. Alternative antibiotics and nitroimidazoles, such as delamanid and pretomanid, have been investigated; however, concerns exist over their resistance and side effects [172]. Experimentation with alternative therapies, such as the utilization of bacteriophages to deliver lytic enzymes, has been proposed as adjunctive therapy for current treatment regimens, but such approaches remain experimental [173]. Host-directed therapies that aim to modulate host factors involved in necrosis may hold promise for improving treatment outcomes in *Mtb* infection. For example, agents targeting cell death pathways, such as necroptosis inhibitors or modulators of mitochondrial function, could potentially reduce necrosis and its detrimental effects on host tissues [168]. Potential adjunctive therapies which target autophagy, apoptosis, and necrosis, outlined in Table 1, may improve the current TB treatment landscape in reducing antimicrobial resistance.

## 6. Conclusions

In conclusion, the intricate balance between *Mtb* infection, immunomodulation, and host cell death via apoptosis underscores the critical role of programmed cell death in controlling tuberculosis pathogenesis. Through apoptosis, infected cells sacrifice themselves, curtailing the replication and dissemination of *Mtb* within the host. This not only limits the spread of the pathogen but also facilitates the removal of infected cells by the immune system, thus impeding disease progression. Without apoptosis-mediated host cell death, *Mtb* would exploit host resources unchecked, leading to an uncontrolled proliferation and exacerbation of infection, preferably through necrosis. Furthermore, apoptosis plays a pivotal role in shaping the immune response, facilitating antigen presentation, and orchestrating the clearance of *Mtb*-infected cells. Therefore, understanding the mechanisms governing apoptosis during *Mtb* infection is paramount for developing effective therapeutic strategies to combat tuberculosis. A better understanding of host cell death pathways may offer promising avenues for the development of novel interventions aimed at bolstering the host’s defense mechanisms against *Mtb*, ultimately advancing efforts towards tuberculosis control and eradication.

While apoptosis-mediated host cell death is a crucial defense mechanism against *Mtb* infection, it has several potential side effects that can impact the human body. While apoptosis is generally a non-inflammatory form of cell death, excessive or dysregulated apoptosis [174] can lead to significant side effects, including tissue damage, inflammation, and the disruption of granuloma integrity, leading to the dissemination of *Mtb* [175]. Additionally, virulent strains of *Mtb* have evolved mechanisms to subvert apoptosis, leading to macrophage necrosis [28,176] and, thus, the release of cellular contents and inflammation. Persistent inflammation can exacerbate tissue damage and contribute to disease progression [151,177]. Lastly, apoptotic cells can sometimes be less effective in presenting antigens to T cells compared to cells that undergo other forms of cell death, which can impair the activation of specific immune responses necessary for controlling *Mtb* infection [178]. Understanding these side effects is important for developing therapeutic strategies that can modulate apoptosis and improve outcomes in tuberculosis.

## Figures and Tables

**Table 1:** List of potential and existing therapies with their associated pathway and targeted mechanism.

Therapy	Pathway	Mechanism
Rapamycin, histone deacetylase inhibitors	Autophagy	Inhibition of mTOR
Metformin	Autophagy	Activation of AMPK
Transcription factor EB	Autophagy	Regulates autophagy gene expression
Lysosomotropic agents	Autophagy	Enhance lysosomal acidity
Nitazoxanide	Autophagy	Activation of mTORC1 inhibition
Vitamin D	Autophagy	Synthesis of CAMP
BH3 mimetics	Apoptosis	BLC-2-mediated apoptosis
Nutlin-3	Apoptosis	Activation of p53
NSAIDs, corticosteroid, zileuton, desipramine	Necrosis	Eicosanoid modulation
Phosphodiesterase inhibitors	Necrosis	TNF reduction

## Data Availability

No new data were created or analyzed in this study. Data sharing is not applicable to this article.
